# Transfer and transition referrals of patients with intellectual disability from children's services to adult community learning disability teams

**DOI:** 10.1192/bjo.2021.307

**Published:** 2021-06-18

**Authors:** Jalil-Ahmad Sharif

**Affiliations:** Solent NHS Trust, Imperial College London

## Abstract

**Aims:**

The audit aimed to assess if patients under the care of children's services in Wessex were transferred at the appropriate age and whether transition referrals to Community Learning Disability teams (CTLD) occurred timely. It also aimed to look at how many patients underwent transitions in a three month period, and if their transition support plan (TSP) was completed. A transition support plan should include chronological information on psychopharmacology, psychotherapy, and social support measures. Patients should be referred between the ages of 17–19 but require a justification after 18 years of age.

**Method:**

The BI team was contacted to provide all IDs for patients referred within a three month period between the ages of 17–19. The BI team provided 42 patients with their ID. Patients discharged from services within a short time span were excluded for the following reason: inappropriate referral (9pts), discharged after 1st assessment (6pts), internal discussion (6pts), only referred to Autism team (4pts), moved out of area (1pts). From the initial 42 patients, 16 patients were analysed using the collection tool.

**Result:**

4/16 had a TSP, and only two had a complete TSP and transitioned in another trust and were inter-team referrals.

CAMHS services referred 1/16 patients.

Psychotropic medication was prescribed to 12/16 prior to or on time of referral, but only two patients had a complete psychotropic medication history.

8/16 patients' referral was commenced prior to their 18th birthday, and no information was provided for delay in transfer.

Health records did mention psychotherapy, but apart from 2/16 TSP records, no additional information was available on the modality.

**Conclusion:**

Patients with Intellectual Disability face challenges when transferring from children to adult services. Insufficient referral information may have a detrimental impact on patients wellbeing and long-term care.

Access to a patient's chronological journey through the different children's services allows Adult CTLD health professionals to provide effective care. Historical psycho-social and pharmacological interventions provide a reference point for future interventions.

Concerns included: limited information on most TSP regarding psycho-social and psychotropic treatments, lack of access to CAMHS/CHYPS paperwork and ineffective inter-trust communication for transition patients.

This project highlighted the average number of transition cases in 3 months. It led to changes to the transition pathway, as awareness was raised in trust and CCG meetings to improve patient outcome. CTLD created the new role of transition facilitators to support children's services. They sit in meetings before patients transition referrals.

